# Neural correlates of bilateral proprioception and adaptation with training

**DOI:** 10.1371/journal.pone.0299873

**Published:** 2024-03-15

**Authors:** Sebastian Rueda Parra, Joel C. Perry, Eric T. Wolbrecht, Disha Gupta

**Affiliations:** 1 Department of Electrical Engineering, University of Idaho, Moscow, Idaho, United States of America; 2 Stratton Veterans Affairs Medical Center, Albany, New York; 3 Department of Mechanical Engineering, University of Idaho, Moscow, Idaho, United States of America; 4 Department of Electrical and Computer Engineering, University at Albany, State University of New York, Albany, New York, United States of America; Kennedy Krieger Institute/Johns Hopkins University School of Medicine, UNITED STATES

## Abstract

Bilateral proprioception includes the ability to sense the position and motion of one hand relative to the other, without looking. This sensory ability allows us to perform daily activities seamlessly, and its impairment is observed in various neurological disorders such as cerebral palsy and stroke. It can undergo experience-dependent plasticity, as seen in trained piano players. If its neural correlates were better understood, it would provide a useful assay and target for neurorehabilitation for people with impaired proprioception. We designed a non-invasive electroencephalography-based paradigm to assess the neural features relevant to proprioception, especially focusing on bilateral proprioception, i.e., assessing the limb distance from the body with the other limb. We compared it with a movement-only task, with and without the visibility of the target hand. Additionally, we explored proprioceptive accuracy during the tasks. We tested eleven Controls and nine Skilled musicians to assess whether sensorimotor event-related spectral perturbations in μ (8-12Hz) and low-β (12-18Hz) rhythms differ in people with musical instrument training, which intrinsically involves a bilateral proprioceptive component, or when new sensor modalities are added to the task. The Skilled group showed significantly reduced μ and low-β suppression in bilateral tasks compared to movement-only, a significative difference relative to Controls. This may be explained by reduced top-down control due to intensive training, despite this, proprioceptive errors were not smaller for this group. Target visibility significantly reduced proprioceptive error in Controls, while no change was observed in the Skilled group. During visual tasks, Controls exhibited significant μ and low-β power reversals, with significant differences relative to proprioceptive-only tasks compared to the Skilled group—possibly due to reduced uncertainty and top-down control. These results provide support for sensorimotor μ and low-β suppression as potential neuromarkers for assessing proprioceptive ability. The identification of these features is significant as they could be used to quantify altered proprioceptive neural processing in skill and movement disorders. This in turn can be useful as an assay for pre and post sensory-motor intervention research.

## 1 Introduction

Movement is both a fundamental necessity for survival and a means for creative, linguistic, and athletic expression. Tasks like tying our shoes, playing a musical instrument, or kicking a soccer ball require accurate and dexterous control of our bodies. Although movement has been studied from multiple perspectives (e.g., kinematics, kinetics, muscle activation patterns, etc.), our understanding of the complex use and integration of real-time sensory (afferent) feedback and input from prior experiential learning (practice and memory) toward movement planning and execution is limited [1,2]. The cohesive integration of sensorial information from different sources is a vital component of movement planning [1,3]; apart from vision, proprioception is another type of this sensorial information. Proprioception, denoting "the perception of one’s self” in Latin, is used to estimate the position and movement of body segments [4].

Damage to brain regions responsible for muscle control leads to impaired movement. Similarly, but less studied, damage or deterioration of proprioceptive sensing and/or processing may also lead to impairment [5–7]. The latter complicates the assessment process of motor deficiencies, making it difficult to know the true source of impairment. As a result, contributions to impairment from proprioceptive losses may be overlooked or underrepresented during clinical evaluation. It is known that proprioceptive accuracy deteriorates with age [8,9], potentially contributing to increased proneness to accidents in the elderly [10]. Proprioceptive and somatosensory deficits have also been related to functional deficits in people with movement disorders in hemiplegic cerebral palsy [5], Parkinson’s disease [11], and in stroke [7]. Additionally, these deficits predict therapy gains in chronic stroke patients [12]. A deeper understanding of proprioception as a contributor to motor control is essential for formulating new therapeutic approaches, developing tools, and designing interventions capable of maximizing motor functional gains in people with movement disorders.

Neural mechanisms in proprioception are under active investigation using diverse neuroimaging modalities, such as functional magnetic resonance imaging (fMRI) [8,13–21], positron emission tomography (PET) [13,22] and electroencephalography (EEG) [23–25], complemented by insights from behavioral results [26–29]. These studies aim to assess: 1) brain regions and rhythms that correlate with proprioception, and 2) changes in proprioception-related activation after a central nervous system injury or a movement disorder. In individuals with intact proprioception, processing of this information activates contralateral sensorimotor regions, particularly the parietal cortex [17,20,25,30] and contralateral posterior parietal cortex (PPC), as indicated by both fMRI [15] and EEG studies [25]. In individuals with injuries, such as post-stroke, lesions in multiple brain areas, including the right supramarginal gyrus [31], and parietal operculum (the secondary somatosensory cortex) [17], have been linked to upper-limbs proprioceptive deficits.

Typical proprioception paradigms studying activation of brain regions often use passive, illusive movement [13,16,19,20,24,32], or active movement in positional replication tasks [18,25,30,33]; with most of these studies using memory-based unilateral tasks [18,30,33], where participants replicate a perceived position from memory with the same hand [34,35]. However, daily activities, mostly bimanual, require bilateral integration of proprioceptive and sensory information from multiple sensory sources. Brain activation patterns in bilateral proprioceptive tasks, in which participants match positions or joint configurations of a concurrent perceived target with the other hand [34,35], are less explored compared to unilateral tasks. However, similar activation patterns to those in unilateral matching replication have been observed for upper [20,30] and lower extremities [18]. Behavioral results from bilateral tasks reveal asymmetries in proprioceptive performance favoring the non-dominant limb [26,27,36,37], which supports evidence of enhanced right hemisphere activation related to proprioceptive processing in right-hand dominant people [14,16,25,30]. Non-dominant limb increased proprioceptive acuity has been linked to the stabilization role of this limb during bimanual tasks [38–40].

Research on sensorimotor brain rhythm modulation in bilateral and unilateral proprioceptive tasks is limited. Investigating physiological aspects of proprioception using EEG during active moments presents challenges due to the spatial resolution of EEG, resulting in mixed activity from close cortical areas responsible for movement and sensation [41,42]. Despite these challenges, careful experimental design and clever comparison of features can help the study of these neural correlates. Marini [25] demonstrated evidence of mu (μ) power modulation during a unilateral proprioceptive-memory task, yet studies on processing of proprioceptive information during bilateral tasks are lacking. Further, the involvement of other frequency bands, such as the beta band (β), known for participating in several sensorimotor and cognitive processes [43,44], remains an unexplored aspect for these tasks.

Skill training, such as in playing musical instruments (piano, guitar, etc.) presumably optimizes proprioceptive processing or limb position sense [45,46], especially bilateral proprioception—understanding the position of one limb relative to the other—, and motor processing [47]. Proficiency in playing instruments relies on efficiently understanding hand positions relative to the other, the body (intrapersonal), and the instrument (extrapersonal) [48]. For example, young pianists exhibit enhanced wrist proprioception [46]. String instrument players show changes in the motor cortex hand representations [49], and changes in white matter architecture, associated with more efficient processing of sensorimotor information [50]. These adaptations contribute to faster reactions to sensory stimuli and better integration of information from multiple sensory sources [47].

Considering these insights, we developed an EEG study to examine and compare neural correlates during a simple, active movement task involving bilateral intra-personal concurrent targets (always present in the workspace without relying on memory). Two distinct cohorts of participants were recruited, healthy adults (Controls) and those trained in playing musical instruments (Skilled). This study aims to understand adaptive mechanisms of sensory information processing attributable to dexterous training, and differences in power modulation for these groups related to multi-source sensory integration. This is done by assessing oscillatory brain activity associated with bilateral distance matching in the μ (8–12 Hz) and low-beta (12–18 Hz, low-β) band, subset of beta band known to be related to upper extremity control and afferent signal processing [51].

Participants performed repetitions of a hand-distance-matching task where the non-dominant (ND) hand (known for increased proprioceptive accuracy,[26]) set target distances to be matched with the dominant hand, restricted to a single dimension (distance from the body). This task was performed with and without visual confirmation of the target distance set by the guiding hand, hereafter known as Bilateral Distance Matching (BDM) and Bilateral Distance Matching with vision (BDM-v). These tasks sought to capture information about setting a perceived (proprioceptive or visuo-proprioceptive) target and translating it into a motor command executed by the opposite hand. Additionally, participants perform a reaching task (No Target Distance, NTD) without a proprioceptive target to match, allowing discrimination of electrophysiological differences related to bilateral proprioceptive matching during active movement versus active movement alone. In all the experimental tasks, participants move their hands in a constrained workspace while wearing a) motion tracking sensors on their hands that provide precise and dynamic movement locations, used to compute distance mismatch in proprioceptive tasks as matching error; and b) EEG electrodes on the scalp for simultaneously recording their electrical brain activity. Vision of the matching hand is occluded in all repetitions of NTD, BDM and BDM-v, while vision of the guiding hand is occluded in BDM and not in BDM-v. All movements are active and time-limited but allow the participants to decide important aspects of movement (pseudo-self-paced), with no assistance from a robot or other external device.

We hypothesize that bilateral proprioception matching will involve modulation of contralateral (with respect to the matching hand) μ band power (as seen in [25] for unilateral tasks) and low-β band power (involved in sensorimotor processes for upper extremities, [43,44,52]). The effect of visuo-proprioceptive integration will be represented by modulation of μ and low-β band power [14,15,25,53], and will be accompanied with smaller proprioceptive errors [1,53]. In behavioral assessments we expect the *Skilled* group to have a higher proprioceptive accuracy (lower distance error) compared to the *Controls* group [46]. Moreover, their neural correlates will be significantly different from the Control group.

## 2 Methods

### 2.1 Participants

A group of 20 healthy individuals (6 females, 14 males; mean age 35.1 ± 16.5 years) participated in the study. All participants were right-handed (self-reported) and had no known neurological disorders at the time of the study. Recruitment was performed on the University of Idaho Campus with recruitment including people from the School of Music. Participants naive to musical instrument playing, or with no history of consistent musical instrument practice in the last 5 years (self-reported) were grouped as the ‘Control group’ (n = 11, 3F/8M, 32.2 ± 15.4 years of age); while those with considerable (professional) musical training in string instruments and piano were grouped together as the ‘Skilled group’ (n = 9, 3F/6M, 38.6 ±18.0 years of age). Participants in this group reported regular practice in the last 5 years, averaging 12.6 ± 5.8 hours of weekly practice. The Skilled group were adept at piano (n = 8) or guitar (n = 1), with an average music experience of 30.9 ± 18.9 years. Information about these participants is presented in Supplementary Information 3 (S1 Table). These instruments were selected due to the important bilateral proprioception component required to play them proficiently [48]. The experiment was performed at the Integrated Research and Innovation Center at the University of Idaho and approved by the Institutional Review Board of the University of Idaho (#21–191). All participants provided written informed consent prior to participation in the study.

### 2.2 Experimental setup

The goal of the experiment was to determine the neural correlates of proprioception during bilateral hand distance matching tasks. For this, the participant is prompted via unique auditory cues to make non-dominant (left-) hand movements to set a target (horizontal distance from the body), and to match this horizontal distance with the dominant (right-) hand. During the experiment, we recorded a) brain signals, with a non-invasive full-head EEG acquisition system [54], and b) precise hand movements with a camera-based motion tracking system [55].

#### 2.2.1 Workspace

**Fig 1:**
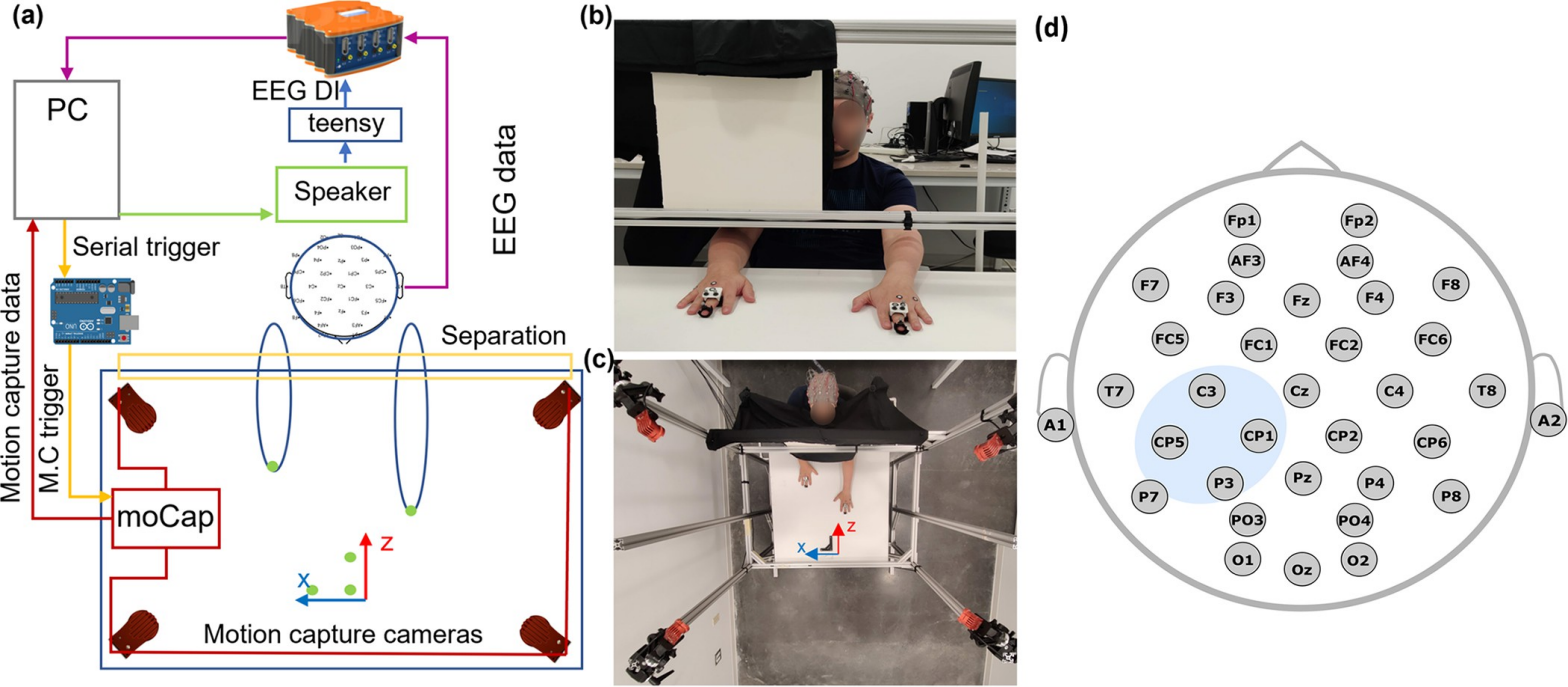
Experimental setup. a) Data collection hardware schematic for experimental setup. b) Experimental workspace front view, partial vision occlusion apparatus and motion capture marker frames shown. c) Experimental workspace, top view. d) EEG montage and selection of spatial features. Blue region represents the contralateral sensorimotor area.

Participants were seated comfortably in a chair, centered in front of a table that delimited the workspace. The proximity of the chair to the 91x91x72.5 cm (width x depth x height) table allowed equal range of motion for both arms within the workspace (Fig 1B and 1C). The view of the workspace was occluded by a retractable structure with dark cloth that minimized perception about the position of their limbs in space, ensuring that participants would not get information about their limb positions based on touch between their arm and the cloth. This cloth draped around the participant’s neck (Fig 1B and 1C) and allowed rapid switching between experimental modalities (vision occluded/partially occluded). The experiment was conducted in a space with controlled lighting and sound.

#### 2.2.2 Motion capture data acquisition

**Fig 2:**
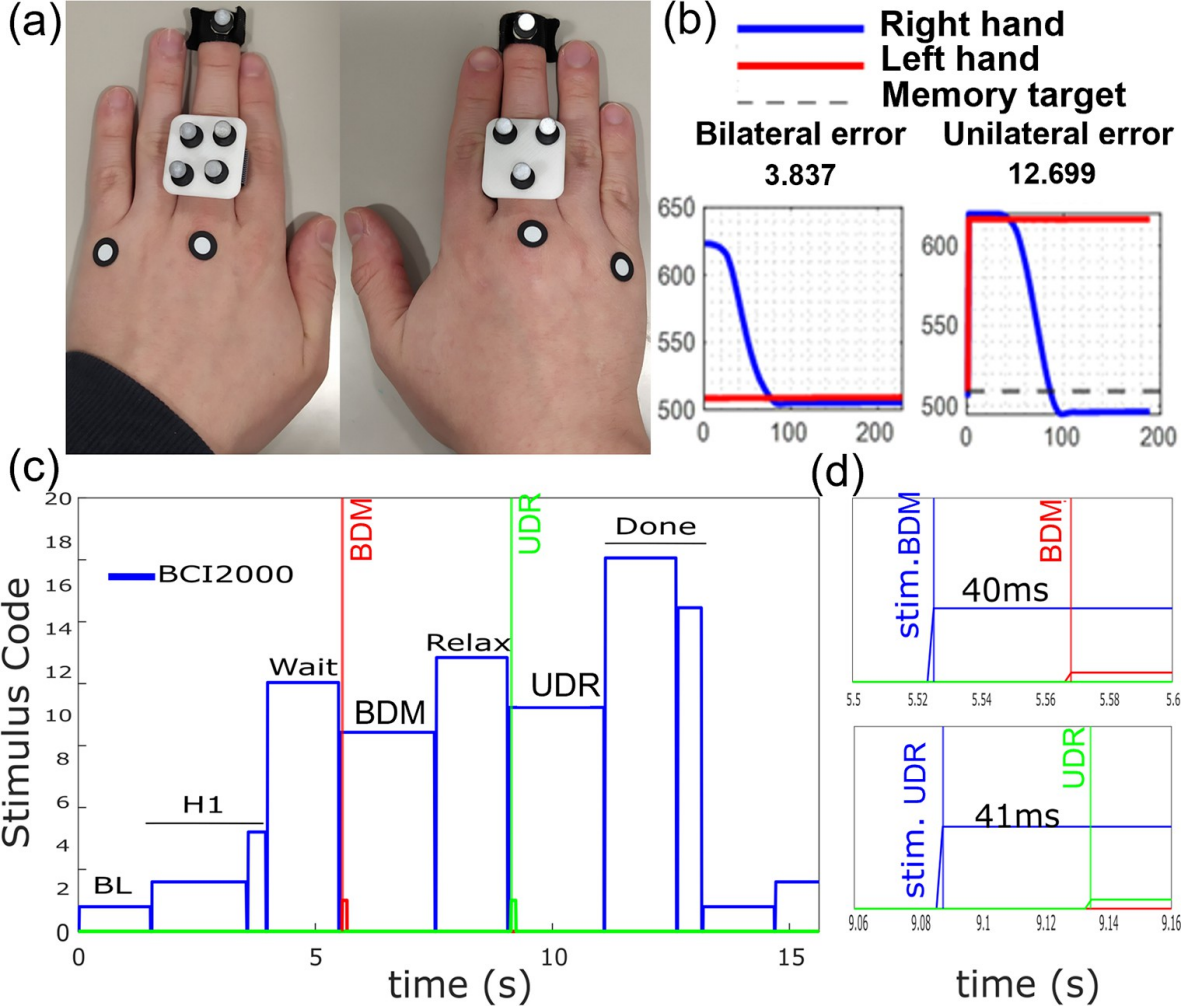
Marker location and proprioceptive matching and replication errors. a) Marker location on right (dominant) and left (non-dominant) hands. b) Example of proprioceptive matching error calculation for bilateral distance matching (left) and unilateral distance replication (right) tasks. c) Stimulus presentation and hardware retriggering. d) Delay of hardware retriggering versus BCI2000 stimulus.

A 4-camera motion-tracking system (Optitrack Flex-13 cameras with Motive software) [55] was used to capture precise hand movements during various tasks of the experiment. An aluminum structure with 4 posts around the experimental table held each camera (Optitrack Flex 13, 120 frames per second) at a height of 115 cm from the table’s surface. Cameras tracked reflective markers that were located on the participant’s hands. The position of each marker was given with respect to a coordinate frame on the table (x-axis positive to the right, y-axis positive up, z-axis positive into the subject, see Fig 1A and 1C). A mean tracking error of 0.235 mm was observed during calibration. We used three sets of reflective markers, placed on the dorsum of both hands of the participant (shown in Fig 2A). These include: a) one marker on the tip of the middle finger of each hand, b) a unique configuration of 3–4 markers (3 for the dominant hand, 4 for the non-dominant hand) on the base of the phalanges, fixed to a small custom 3D printed square tile with depressions for the spherical markers, worn as a ring, and c) adhesive markers on the dorsal surface of two MCP joints (middle and little finger). In the Optitrack Motive software, all 3 marker sets on each hand are connected as a rigid body, allowing more accurate tracking of the fingertip, which is used to indicate the extension distance of the hands in this study. The onset of each trial (i.e., the moment in time when the participant heard the auditory cue to move the hand) was used to trigger motion-capture data recording, and to mark the data with a unique identifier state sent by the BCI2000 software via the *appConnector* module [56].

#### 2.2.3 EEG setup and data acquisition

32-channels of referential EEG were recorded at 512Hz, using a g.Hiamp [54] and BCI2000 data acquisition software [56]. Active Ag/AgCl-ring electrodes were placed using positions from the 10–10 standard International EEG montage (Fig 1D), with ground at anterior frontal zero (AFz) and reference at the left earlobe. Electrode impedances were maintained below 20kΩ. Participants were cued to move hand(s), pause and retract hand(s) via auditory cues (300 ms duration, synthesized female voice commands or tones of 500 and 1500 Hz), presented with the BCI2000 Stimulus Presentation module [56]. The speaker was placed centrally behind the participant, to avoid any sound location bias. Additionally, we used a hardware re-triggering approach to ensure that the stimulus onset precisely recorded the moment when the stimulus was heard, rather than when it was scheduled to be played (Fig 2C and 2D). This approach uses auditory cues that are transmitted as stereo signals, with the left channel carrying the spoken (or tone) cue to the speaker; and the right channel carrying a tone burst (75 ms duration, [500–1500] Hz freq. range) that is captured by a microcontroller (Teensy 2.0, ATMega32U4). The microcontroller instantaneously captures and converts the incoming tone into a digital TTL pulse, and transmits it to the EEG acquisition system via a BNC cable (Fig 1A). This is recorded as a digital I/O trigger channel in sync with the EEG data. This real-time auditory signal conversion was made possible with the help of a modified version of the Audiomath library [57] and the Teensy 2.0 USB-microcontroller board. We used unique tones to record the onset of different conditions.

#### 2.2.4 Auditory cues

Auditory cues were provided with the Stimulus presenter app module of BCI2000 [56]. The auditory cues prompted the participants during the experiment with either synthesized voice commands or tones. Unique tones indicated bilateral matching phase (frequency 500 Hz with duration 75 ms) and unilateral distance replication phase (frequency 1500Hz with duration 75ms).

### 2.3 Paradigm design

Our hand position matching tasks are similar to those used in studies that measured proprioceptive accuracy and hand motion [34,58,59]. Participants performed bilateral and unilateral proprioceptive tasks presented sequentially, with and without partial visual occlusion of the workspace. In this paper we will only analyze results from bilateral matching tasks (BDM and BDM-v). Both groups of participants also completed another vision occluded task in which no matching was performed, this task consists of dominant (right) hand motion only and was used to reference the analysis of neural correlates of bilateral proprioception, with the aim of removing neural correlates of active motion.

**Fig 3:**
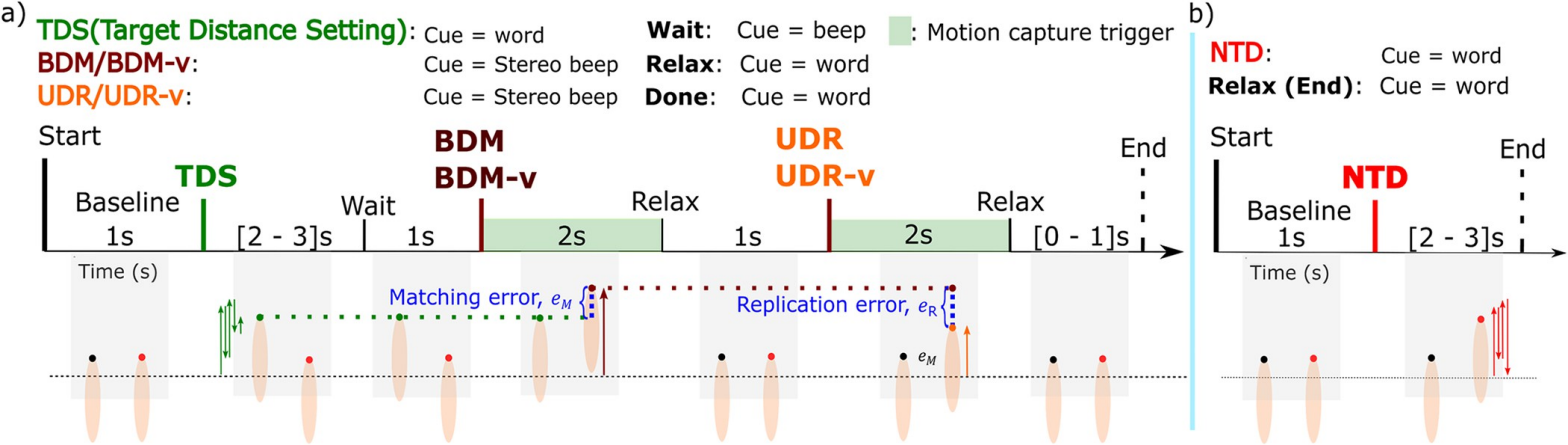
Timing for one trial of the experimental paradigm. a) Timing schema for bilateral (BDM, BDM-v) and unilateral (UDR, UDR-v) proprioceptive matching tasks. b) Timing schema for the movement without proprioceptive matching (NTD) task.

In the proposed bilateral hand distance matching task (BDM and BDM-v), participants were asked to accurately pair the perceived reach (horizontal) distance of the non-dominant hand with the dominant hand. In the unilateral distance replication task (UDR and UDR-v), the goal was to recreate the reach distance of the dominant hand from memory after it had recently been held and retracted from a location. This distance resulted from the attempted matching in the bilateral matching task. For the visual modality, participants are allowed to only see the guiding hand in the target distance setting phase (TDS, Fig 3A). The position indicator used to assess placement accuracy was the marker on the tip of the middle finger.

At the start of each trial, both hands were placed (prone) at the reference (or home) position (arms opened at shoulder’s width with the palms resting at the edge of the table, shown in Fig 3A, Baseline phase). The first cue (spoken word “left”) indicated non-dominant hand movement (Fig 3A, TDS phase). For this phase participants had been instructed to repeatedly extend and retract their non-dominant (left) hand straight out at their own pace, while keeping their hand above the table without touching it. A randomized period between 2 and 3 seconds after the “left” cue, a “wait” cue instructed them to rest that hand on the table while maintaining the z position (horizontal distance) at the time of the cue. One second after the “wait” cue, a unique tone instructed them to extend their dominant hand (occluded from view and resting on the table) to try and match the fingertip extension of their non-dominant hand (Fig 3A; BDM, BDM-v). They had 2 seconds to perform this matching, before they were cued to relax both hands bringing them back to the home position. This was followed by a second unique tone, cueing them to replicate the extension distance of the dominant hand (occluded from view, relying on unilateral proprioception) from the previous bilateral matching task (Fig 3A; UDR, UDR-v). After allowing 2 seconds for replicating, the “relax” command directs the participants to bring their dominant hand to the home position. The trial ends with a randomized inter-trial rest period of 1 to 2 seconds.

Task modes with and without vision were randomly interspersed as blocks of 11 trials each. A total of 132 proprioceptive trials were collected in a single session with 12 blocks of 11 trials; 66 repetitions in total for each task in both modalities (vision occluded, and vision not occluded). For these tasks, the dominant hand was always kept occluded from view. The non-dominant hand was either made a) visible (in 50% of the blocks; BDM-v and UDR-v tasks), or b) kept occluded (in BDM and UDR tasks). In addition, we also interspersed 3 blocks of 22 short trials (66 repetitions), with only dominant hand movement without proprioceptive matching (NTD, occluded from view), corresponding to Fig 3B, for use as a reference in the analysis.

The experiment was performed in a single session of 90 minutes, with rests between blocks to ensure engagement and motivation. Three minutes of practice trials were performed prior to commencing data collection, allowing participants to be acquainted and comfortable with each condition and its cues.

### 2.4 Analysis

#### 2.4.1 Motion capture data analysis

Trajectory data for each hand location was sorted by experimental task (BDM, BDM-v; first green shaded time periods in Fig 3A). Repetitions contaminated by artifacts were removed by visual inspection. Trials with erroneous movement or with motion tracking system noise were flagged and removed. An average of 1.7 ± 2.8 repetitions were removed from the BDM task and 1.6 ± 2.8 were removed from the BDM-v task for all participants.

To quantify participant’s proprioceptive accuracy and assess their ability to perceive distance discrepancies between hands (guiding and matching hands), proprioceptive matching errors were derived. These errors were computed using averaged samples of extension distance (z position in Fig 1C) during the last 24 samples (100 ms) for each trial. The analysis of errors was confined to one dimension to simplify the analysis of EEG signals.

For the bilateral matching task (tasks BDM and BDM-v) matching error, *e_M_*, was computed as the absolute value of the horizontal distance difference between the left (non-dominant, target) and right fingertips (Fig 3A). Global proprioceptive errors were computed as the median error across trials for each experimental task (BDM, BDM-v).

#### 2.4.2 EEG data analysis

Data was processed in Matlab (R2019a, The Mathworks, Natwick, MA), using EEGLAB [60] and custom functions. EEG data was down-sampled to 256 Hz, high-pass filtered with a cutoff frequency of 0.5Hz (Hamming Windowed Sinc FIR filter with automatic estimation of filter length [60]), followed by line noise removal (60Hz and harmonics, using the CleanLine plugin [61]). EEG channels were inspected and flagged for removal if they contained more than 5 seconds of flat activity (no channels were flagged from this experiment). Data was then re-referenced to the common median reference, followed by removal of transient high-amplitude artifacts using an objective artifact subspace reconstruction method [62]. Data was then re-referenced to the common median reference again, prior to further denoising.

Next, we used independent component analysis–AMICA [63], and EEGLAB IClabel plugin [64] to automatically identify and remove signal components that contain artifacts. The denoised data was epoched (-1 to 2 seconds) into trials, with the hardware re-triggers indicating time zero for each experimental task. Lastly, at the trial level, we used a custom function to automatically remove trials with artifacts based on pre-specified statistical metrics (Average amplitude and Kurtosis across trials). On average for all participants, 2 ± 1.6 repetitions were removed for the NTD task, 2 ± 1.9 repetitions were removed from the BDM task, and 1.45 ± 1.4 repetitions were removed for the BDM-v task.

#### 2.4.3 Event Related Spectral Perturbation (ERSP)

We computed ERSPs [65] to analyze the spectral content of the EEG data of electrodes overlapping the contralateral (left) sensorimotor area [13,51] and assess its relationship with proprioception and movement. The Morlet wavelet convolution [41] was used to compute time-frequency decomposition using kernels from 2 to 35Hz, with 4 cycles for lower frequencies and 10 cycles for higher frequencies. Resulting time-frequency maps were baseline normalized using the gain model (i.e., a division with the baseline mean power), baseline period being -600 to -100 ms with respect to the start of the trial. Thus, the ERSP was computed as the average event-related variation across trials (in dB) compared to the respective baseline [65,66].

The extracted features are the mean ERSP over time ranges of interest, frequency bands of interest (μ and low-β bands) and regions of interest (ROIs) (Fig 1D). There is evidence of μ band modulation in unilateral proprioceptive tasks [25] and the impact of the β band in processing of sensory information [43,44] with the low-β playing a significant role in upper-limb sensorimotor activity [43]. Movement termination stage (offset, 400 to 800 ms) was the period of interest for the analysis since it reflects the end of the extension distance matching process. This interval was selected prior to the data analysis and was based on a typical average movement response after an auditory command [67–69], for example, see [25]. These features were extracted for all the experimental tasks (NTD, BDM, BDM-v). Specific combinations of features regarding experimental tasks, time ranges of interest, groups, and ROIs were used to address different research questions.

### 2.5 Statistics

Two-way mixed model ANOVAs were used to perform comparisons of proprioceptive errors, distance targets set by the non-dominant (ND) hand, and ERSP features for the μ and low-β bands. The within factor was the task (NTD and BDM, or BDM and BDM-v for ERSP features, and BDM and BDM-v for kinematic variables), while the between-factor was the group (Controls and Skilled). ANOVA models were fit after testing for normality (Shapiro Wilks Test).

The main effect of the group (between) variables was assessed using non-paired t-tests (comparison of same task, between groups), and within effect was assessed using a paired t-test (comparison of the same group, between tasks). Pairwise post-hoc comparisons were corrected for multiple corrections using the False Discovery Rate (FDR) method [70]. We considered FDR adjusted p-values < 0.05 (adj-p) to be significant. For effect size calculation, Hedges’ g (g) was used due to the sample size (computed as the corrected difference of means divided by the pooled standard deviation). Effect size is defined as very small (0.01 to 0.2), small (0.2 to 0.5), medium (0.5 to 0.8), large (0.8 to 1.2), very large (1.2 to 2), and huge (> 2) [71].

## 3 Results

### 3.1 Behavioral results

**Fig 4:**
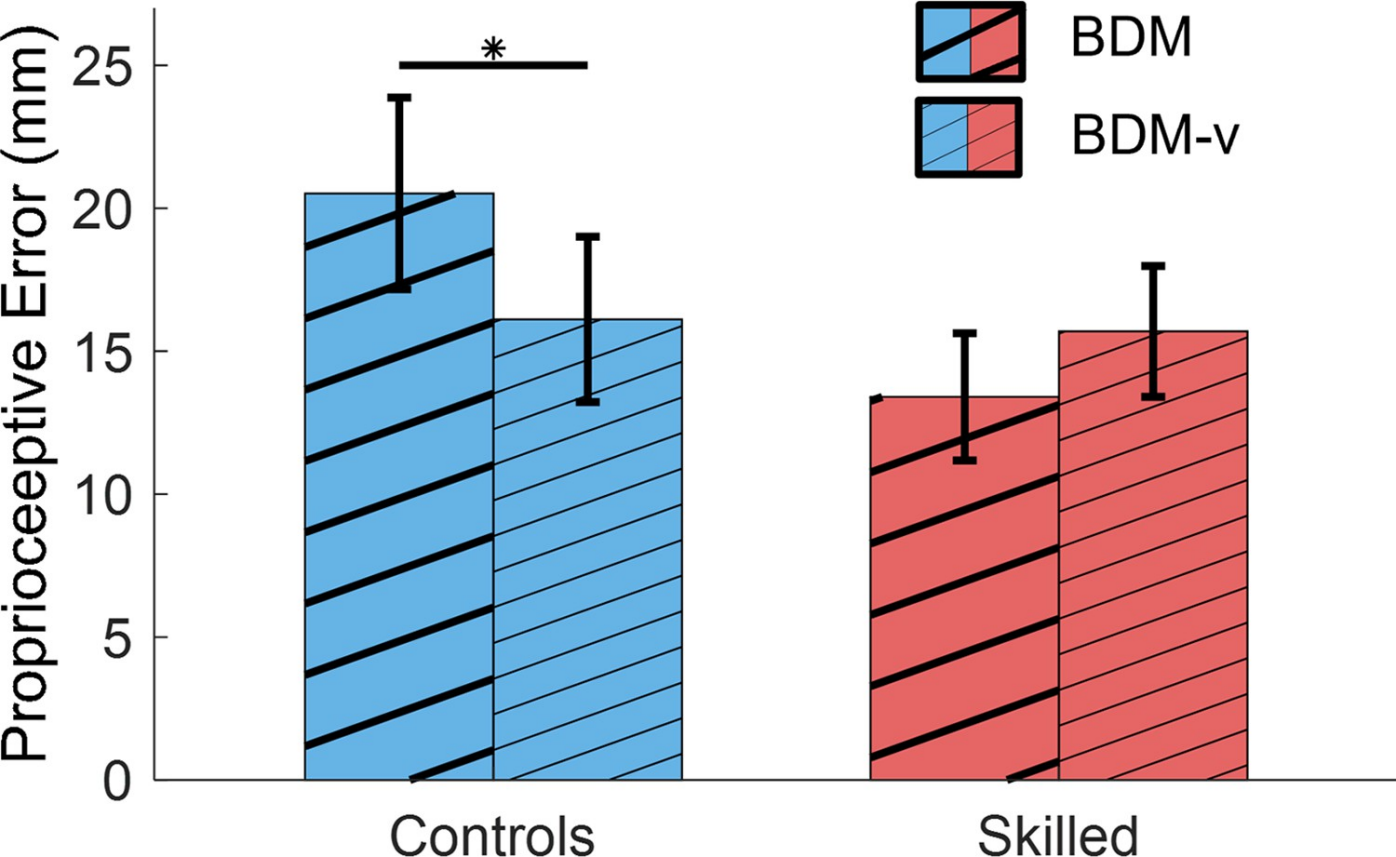
Comparison of proprioceptive groups for bilateral task with and without visibility of the target. The Skilled group shows a tendency to make smaller errors in the bilateral task, while keeping targets closer to the body. The Controls group showed a significant reduction in error with visibility of the target, while the Skilled group did not show a significant change when the target was visible. Significant differences are indicated with a horizontal line and an asterisk.

We tested proprioceptive accuracy, as determined by the proprioceptive errors (*e_M_* in mm), for the bilateral task with and without target visibility (BDM-v and BDM). Fig 4 presents the proprioceptive errors of both groups in both tasks. The mixed-effect ANOVA shows a significant interaction between group and task (F(18,1) = 5.9, p = 0.025) for the comparison of errors derived from the tasks. Subsequent subsections further test these results, based on post-hoc comparisons.

We also analyzed the distributions of target distances. The mixed-effect ANOVA showed no significant interaction between the task and group (F(18,1) = 1.164, p = 0.28). However, a significant main effect of group was identified using a two-way ANOVA (F(18,1) = 10.6, p = 0.002). No significant effect of task was observed (F(18,1,) = 2.22, p = 0.144). The location of the guiding hand with respect to the origin of coordinates from the Motion Capture System in the Z component was (Controls: 484.6 ± 30.6 mm; Skilled: 507.9 ± 14.8 mm) for the BDM task and (Controls: 470.3 ± 32.6 mm; Skilled: 499.0 ± 14.1 mm) for the BDM-v task (greater values indicate positions closer to the body). This suggests that in general, the Skilled group kept their distance targets closer to the body during both tasks.

#### 3.1.1 Effect of music instrument training on proprioceptive error

The subsequent main effect comparison between groups shows no significant effect for the BDM task (adj-p = 0.22, g = 0.81) nor for the BDM-v task (adj-p = 0.94, g = 0.10). However, a trend was noted for the BDM task in which the Skilled group showed smaller and less variant errors (12.41 ± 6.67 mm) compared to the Control group (19.89 ± 11.12 mm) for the bilateral task without target visibility (BDM, Fig 4).

#### 3.1.2 Effect of visual input on proprioceptive accuracy

Subsequent post-hoc within group comparisons were performed to determine the impact of visual input on bilateral proprioceptive ability. We compared (Fig 4) the matching error within groups when the proprioceptive target hand is visible (BDM-v) versus occluded (BDM). The Control group accuracy improved significantly (from 20.52 mm to 16.11 mm) when the target was visible (paired, right tailed t-test, adj-p = 0.02, g = 0.74), whereas the Skilled group did not show a significant change (adj-p = 0.87, g = -0.53). When vision is not occluded (BDM-v), proprioceptive matching errors for the Control and Skilled groups BDM-v are similar (16.11 mm for controls, 15.69 mm for Skilled). This suggests that the Controls group may rely more on vision for proprioceptive accuracy compared to the Skilled group.

### 3.2 Neural correlates of active bilateral proprioception

The neural correlates of proprioception and movement were estimated for μ and low-β bands in the sensorimotor regions of the cortex, for the offset period, as described in the Methods section. The following sections show baseline normalized values expressed in dB, where a suppression in band power, relative to the baseline period (i.e. < 1), is seen as a negative dB value, and vice versa. A larger suppression has a larger negative value.

#### 3.2.1 Movement -with and -without-proprioceptive matching: less μ and low-β band suppression in the contralateral sensorimotor cortex in Skilled group

**Fig 5:**
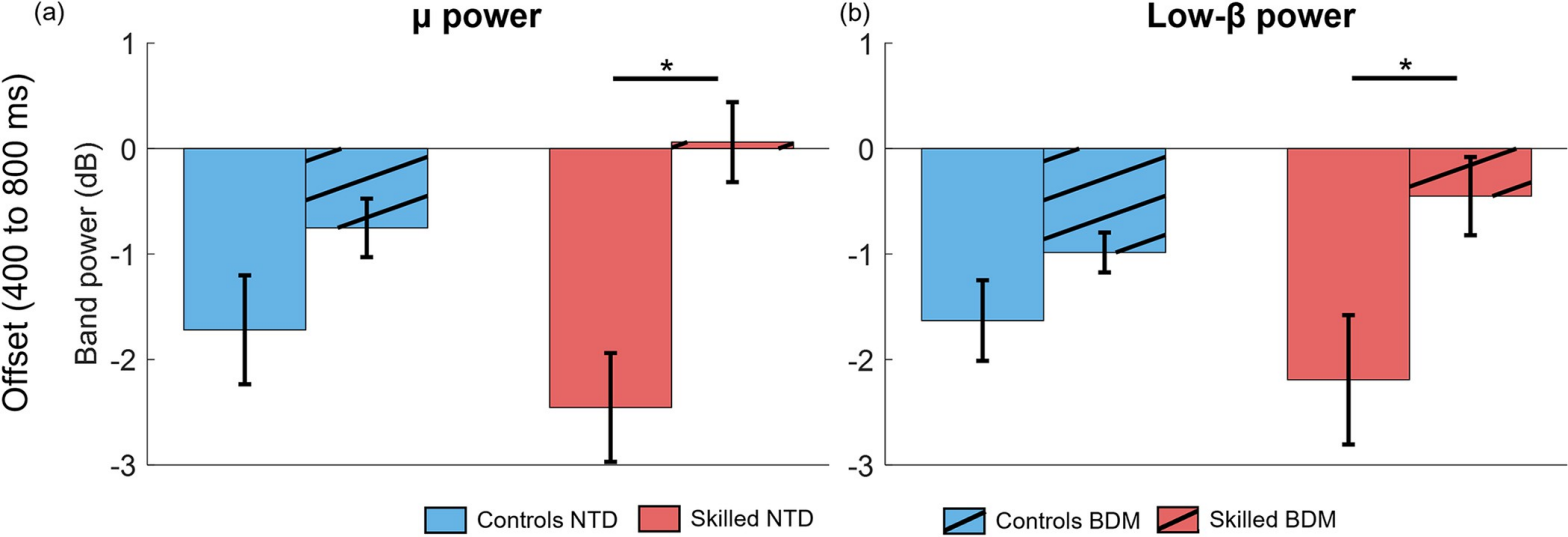
ERSP features (μ and low-β band power) during the bilateral matching task (BDM) and baseline reaches (NTD) for the Controls and Skilled group. a) μ activity at movement offset. b) Low-β activity at movement offset. Significant differences are indicated with a horizontal line and an asterisk.

We assessed the ERSP features for the two groups, Control and Skilled, for the Bilateral task and the task without proprioceptive matching (BDM & NTD)–specifically during the offset period (presented in Fig 5, for μ and low-β bands). A significant interaction was found between the two factors, group (Skilled and Control) and task (BDM and NTD), for μ (F(18,1) = 4.7, p = 0.044). No statistically significant interaction was observed for low-β (F(18,1) = 3.67, p = 0.07). These outcomes were assessed through two-way mixed ANOVA models.

Post-hoc comparisons for the μ band showed no significant differences between groups for the NTD task (p-adj = 0.33, g = 0.63), and the BDM task (p-adj = 0.09, g = -0.81). When comparing this feature between tasks within the same group, no significant difference was found for the Control group (p-adj = 0.08, g = -1.12). However, a significant difference was observed for the group of skilled participants (p-adj = 0.001, g = -2.60), depicted in Fig 5A.

Post-hoc feature comparisons in the low-β band between groups revealed no significant differences for either the NTD task (p-adj = 0.43, g = 0.40) or the BDM task (p-adj = 0.2, g = -0.53). However, a significant distinction was observed in the within-factor comparison for the Skilled group (p-adj = 0.005, g = -1.88), whereas no differences were found for the Control group (p-adj = 0.1, g = -0.83). This suggests less suppression in the group of skilled participants for both frequency bands.

In general, across all participants, we note that the μ and low-β suppression is less during bilateral matching compared to free arm reaches. This implies that proprioception plays a role in modulation of sensorimotor cortical activity. Additionally, these results indicate specific changes in both frequency bands, particularly within the Skilled group.

#### 3.2.2 Effect of proprioceptive target visibility: Contralateral sensorimotor cortex shows μ and Low-β power reversal when target is visible; Controls show a larger reversal

**Fig 6:**
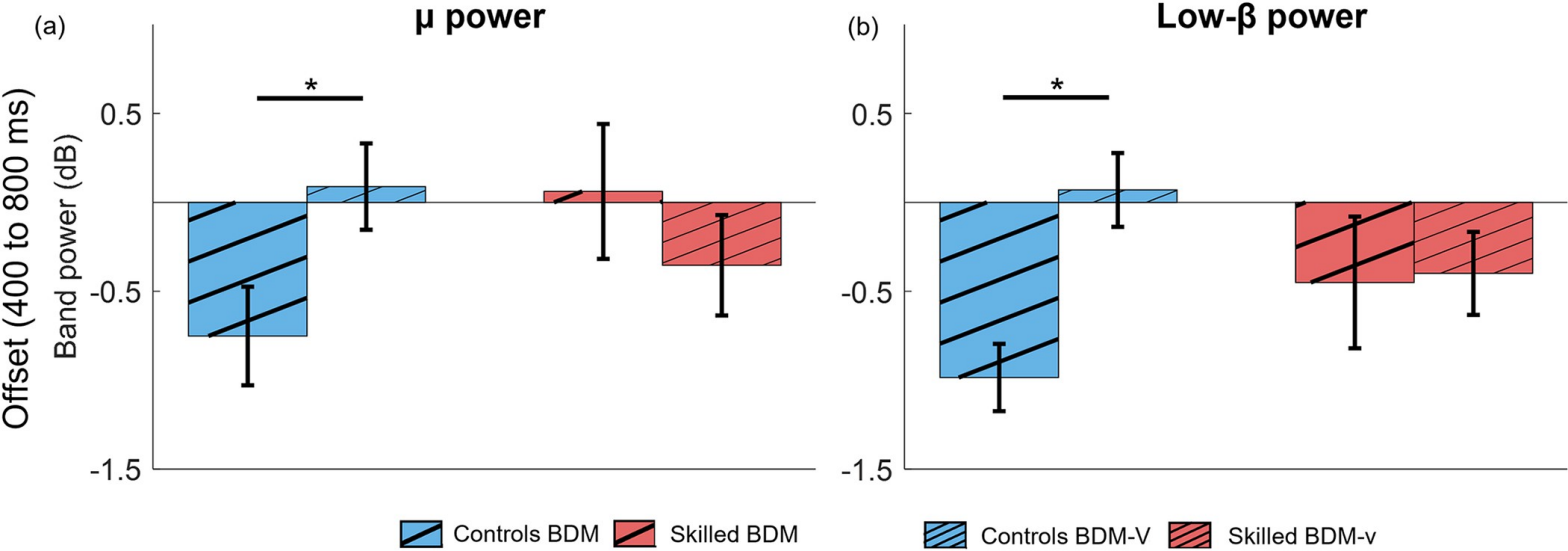
Intra-group comparison of ERSP features for bilateral tasks with and without visual confirmation of the target (BDM-v, BDM) for Controls and Skilled groups. a) μ activity at movement offset. b) Low-β activity at movement offset. Significant differences are indicated with a horizontal line and an asterisk.

Next, we evaluated differences in cortical μ and low-β power between the visual and nonvisual bilateral matching tasks (BDM-v and BDM, as illustrated in Fig 6). In both cases, the two-way mixed models for the μ and low-β bands exhibited statistically significant interactions of factors (μ: F(18,1) = 6.2, p = 0.02; low-β: F(18,1) = 10.8, p = 0.004).

Post-hoc pairwise comparisons indicate no significant effect between the groups for the μ band for the same task (BDM: p-adj = 0.09, g = -0.81; BDM-v: p-adj = 0.25, g = 0.49). However, a significant effect was noted for the difference between tasks within the Control group (p-adj = 0.03, g = -0.97), while no such effect was observed for the Skilled group (p-adj = 0.31, g = 0.48), as shown in Fig 6A.

No significant effects were found for features in the low-β band for the same task between groups (BDM: p-adj = 0.19, g = 0.42; BDM-v: p-adj = 0.15, g = -0.53). When assessing the effect of group between tasks, a significant difference was noted in the Control group (p-adj = 0.0006, g = -1.27), but not in the group of Skilled participants (p-adj = 0.81, g = -0.06).

This outcome suggests greater modulation of sensorimotor cortical power (μ and low-β bands) specifically in the visual task for the Control group, compared to the task without visual feedback. Additionally, no power changes were noted in the low-β band for the Skilled group.

## 4 Discussion

### 4.1 Behavioral results

#### 4.1.1. People skilled (Skilled group) in playing musical instruments showed no enhanced bilateral proprioceptive accuracy compared to the unskilled controls group

Playing a musical instrument requires dexterous movement and complex interaction of proprioception and other sensory modalities [47,48]. Enhanced dexterity seen in skilled people [45] likely reflects the effect of consistent skill practice, which also improves finger strength and individuation [72], and is related to increased proprioceptive acuity [45,46].

In the task where the bilateral target was hidden from view (BDM) (Fig 4), no differences were found between groups. The anticipated increase in bilateral proprioceptive matching accuracy for Skilled participants aimed to highlight a heightened proprioceptive sense of hand position in space, particularly when blindly mirroring hand positions [45,47]. Confirming this effect may necessitate a larger sample size, as a noted trend favoring the Skilled group’s matching accuracy could indicate a small effect that was not discerned with the current data (Fig 4). However, this emerging pattern could also be affected by proprioceptive bias related to specific limb configurations, given that the skilled group consistently placed targets closer to their bodies, a behavior associated with more accurate positional estimations as documented for the elbow [26,28], and knuckles [59]. The Skilled group’s consistent tendency to position their hands closer to the body might stem from their training, suggesting that they try to use familiar positions [48] to favor their proprioceptive accuracy, overseeing instructions that aimed to position targets randomly (see section 2.3).

Additionally, in our experiment participants were strictly directed to exclusively match horizontal distances with the body as a reference point. However, the task lacked control for possible deviations from the horizontal line, and the computation of errors was confined to the horizontal dimension. This specific constraint introduces a potential confounding factor in our results, given the established knowledge that targets closer to the middle line tend to yield more accurate responses [26,28,29,38]. An approach assessing positional matching in two dimensions would provide a more comprehensive understanding of the group’s matching performance, as well as a more accurate depiction of proprioceptive matching differences between groups.

#### 4.1.2 The unskilled controls group uses vision to improve bilateral matching

In the tasks where the bilateral target was visible (BDM-v) the Control group’s performance was closer to the Skilled group (Fig 4). This suggests that people can improve their accuracy in proprioceptive matching of a bilateral hand position when they can see their target hand position, rather than when they are only using their proprioceptive sense [1,25,53]; despite setting more distant targets (i.e., more elbow extension) than the Skilled group. These results support the role of vision as a predominant sensory modality for reaching gestures [15,25,73,74]. When planning movement, vision relates the objective points using an extrapersonal framework for reference, and proprioception provides information about the initial positions of joints [1] using the body landmarks as reference (intrapersonal framework) [59].

On the other hand, the Skilled group did not show a significant difference in proprioceptive accuracy when the target was visible versus when it was not visible. This suggests that such skill training may lead to an improved and efficient integration of multi-sensory inputs. Vision, being still predominant, is perhaps used in rapidly recalling learned proprioceptive patterns that reduce proprioceptive drift, as observed in trained piano players [48].

### 4.2 Neural correlates of bilateral proprioception during movement

Proprioceptive processing has been linked to activity in the sensorimotor region [13,51], where beta oscillations (13–30 Hz) have been correlated with sensorimotor processing and transmission [43,44,51,75]. In particular, the low-beta band (< 20Hz) plays a significant role in upper limb sensorimotor activity [52]. Sensorimotor processing and transmission follow the somatotopic organization [76,77], and is typically observed bilaterally over sensorimotor areas, with a larger involvement of the contralateral sensorimotor cortex [78–81]. The functional role of beta oscillations is debated, but in resting state its presence is considered to mark the ‘status quo’ or state of equilibrium [82,83], and/or sensorimotor integration for motor control [43,44,84], temporal anticipation [85], and error monitoring [86].

Beta oscillation is known to decrease with movement [43,82], referred to as the event related desynchronization (ERD) or beta suppression, and reemerges after movement offset, referred to as event related synchronization [43,51]. Beta suppression is associated more with top-down control, such as releasing inhibition and allowing movement initiation and execution [43,44]. The amount of β suppression has been shown to modulate with aspects of uncertainty in motion, such as timing [87], effector to use [88], and direction of motion [89]; with increased suppression associated to tasks that need more top-down control and vice versa. Such increased top-down control with increased beta suppression can be seen in studies of complex tasks [90], aging [91–93] and Parkinson’s Disorder [94]. Less top-down control and less beta suppression is seen in studies of motor learning that have more automatic or trained movements [95,96].

Our study outcomes align with the previous work discussed above, adding support to these existing models of neural oscillations in sensation and movement and adding evidence further linking proprioceptive processing and beta suppression, as discussed in the following sections.

#### 4.2.1 The Skilled group showed reduced power suppression relative to movement only, compared to the Controls group

The Skilled group of participants had prior guitar or piano training, which has a dominant bilateral proprioceptive element. We were able to test whether the amount of μ and low-β modulation differed with prior proprioceptive skill training when compared to the Controls group. Our findings revealed differences in both bands. Notably, the difference in power suppression between movement only and those with blind bilateral matching was larger for the Skilled group (Section 3.2.1, Fig 5). This result is concordant with the literature that shows increased μ suppression in unilateral proprioceptive tasks [25], and reduced β suppression in cases of reduced top-down control; including less complex tasks [90–92] or after training [95,96]. This is potentially facilitated by other structural and functional changes in the brain of trained instrument players, such as increased cortical representation of hands for string instrument players [49], and fiber connectivity that allows more efficient somatosensory processing [50,97], and multisensory integration [47,98].

#### 4.2.2 Power suppression in skilled people is not affected by target visibility; modulated significantly in Controls

When comparing the neural features in BDM and BDM-v for the Skilled and Control groups (Section 3.2.2, Fig 6), we observe that the μ and low-β suppression is equivalent for the Skilled group in the two tasks, while low-β suppression is significantly reduced for Controls (in some cases increased compared to baseline, Fig 6B). This may indicate that the Skilled group is less affected by target visibility in the bilateral proprioceptive task and can efficiently integrate the visual information with the proprioceptive information for the ensuing hand movement [47,48]. Similar multisensory integration has previously been associated with musical instrument training [47]. Elevated low-β for BDM-v in Controls (Fig 6) may be related to enhanced multisensory integration demanded by the task. Marini et al., [25] showed a similar reduction in μ suppression in tasks that included visual input in a unilateral proprioceptive task. This result also matches their behavioral proprioceptive accuracy in BDM vs BDM-v (Fig 4), where the Skilled group makes equivalent errors in BDM and BDM-v, while the Controls makes slightly larger errors in BDM, which become comparable to the errors of the Skilled group in BDM-v.

## 5 Conclusions

The study presented here investigated the neural features of proprioception during bilateral reaching tasks, where the participants attempted to bilaterally match a reaching distance using proprioceptive information perceived from their ND hand, both with and without target visibility. Two participant groups completed the tasks: a Skilled group with several years of musical instrument training (that includes a bilateral proprioceptive training element), and a Control group with no history of proprioceptive training. We hypothesized that the Skilled group would achieve lower matching errors in the experimental tasks due to their proprioceptive training, and that we would identify neural features related to the observed behavioral differences between the groups.

Contrary to expectations, the Skilled group did not surpass the Control group in bilateral matching errors when vision was occluded, prompting the need for larger sample size, and refined experimental restrictions for further validation. Interestingly, with target visibility, only the Controls group significantly reduced their matching errors, confirming the importance of vision integration in proprioceptive tasks, especially without specialized training.

In the analysis of contralateral sensorimotor features within the μ and low-β bands, our comparisons of μ align with findings presented in the literature for unilateral matching tasks. Additionally, comparisons of low-β band power yield evidence of this band’s crucial role in processing proprioceptive information during bilateral tasks. The modulation of low-β band power during movement for the Control group appears linked to several functional aspects of proprioceptive processing. Power in this band demonstrated modulation relative to task complexity (more suppression with a more complex task), integration of multi-source sensorimotor information (increased power with more converging sources), and proprioceptive uncertainty.

The Skilled group showed similar levels of low-β power during bilateral proprioceptive tasks, with less overall suppression compared to the Controls group. This reduced suppression may be attributable to less top-down control and less proprioceptive uncertainty, a result of enhanced efficiency in multisensory integration resulting from years of proprioceptive motor training. Notably, no differences in low-β power suppression were observed in bilateral tasks with and without target visibility for the Skilled group, in contrast to Controls. This suggests that the role of vision remains dominant after training, but when not available, training can heighten the influence of proprioceptive information while refining the use of low-β power modulation during movement. This finding further highlights the role of low-β activity on the contralateral-sensorimotor cortex in processing and integration of multisource sensory information.

## 6 Limitations and future directions

This study provides valuable insights into cortical power modulation, proprioceptive training, and movement-sensory integration. However, the results presented in this article, especially those of kinematic nature, are limited by having a relatively small sample size and by design constraints, such as restricting movements to only one dimension and not controlling the position of the workspace for the ND (guiding) hand during the tasks. In future studies, it would be useful to a) Replicate the study on a larger sample for better generalizability, b) Explore comprehensive kinematic analysis involving multiple dimensions of space and their relation to neural features, c) constraint the ND target distance using positional references that do not elicit sensory feedback; so participants only set targets within a desired range, and d) Perform the experiment with reversed hand roles to assess the influence of hand dominance on bilateral proprioceptive tasks.

In addition to these, it is relevant to a) expand investigation to include other cortical regions, especially the ipsilateral sensorimotor area (related to matching hand); to gain a broader perspective on sensorimotor processing, and b) Examine how multiple sensory modalities integrate, assessing connectivity, during bilateral proprioceptive tasks.

Addressing these limitations and pursuing the suggested future directions will enhance our understanding of the roles of cortical power modulation and proprioceptive training in movement and sensory integration.

## Supporting information

S1 Table Information about participants in the Skilled group (musicians). (DOCX)

## References

[1] Sarlegna FR, Sainburg RL. The Roles of Vision and Proprioception in the Planning of Reaching Movements. In 2009. p. 317–35. 10.1007/978-0-387-77064-2_16 PMC3709263 19227507

[2] Mostafa AA, THart BM, Henriques DYP. Motor learning without moving: Proprioceptive and predictive hand localization after passive visuoproprioceptive discrepancy training. PLoS One. 2019;14(8):1–19. doi: 10.1371/journal.pone.0221861 31465524 PMC6715176

[3] Holmes NP, Spence C. The body schema and the multisensory representation (s) of peripersonal space. 2006;5(2):94–105. 10.1007/s10339-004-0013-3 PMC1350799 16467906

[4] Sherrington C. The integrative action of the nervous system. J Nerv Ment Dis. 1907;34(12):801–2.

[5] Gupta D, Plains W, Medicine WC, Science C, Hayes H, Haverstraw W, et al. Effect of sensory and motor connectivity on hand function in pediatric hemiplegia. 2018;82(5):766–80. 10.1002/ana.25080 PMC5708868 29034483

[6] Rowe JB, Chan V, Ingemanson ML, Cramer SC, Wolbrecht ET, Reinkensmeyer DJ. Robotic Assistance for Training Finger Movement Using a Hebbian Model: A Randomized Controlled Trial. Neurorehabil Neural Repair. 2017;31(8):769–80. doi: 10.1177/1545968317721975 28803535 PMC5894506

[7] Rand D. Proprioception deficits in chronic stroke—Upper extremity function and daily living. PLoS One. 2018;13(3):1–10. doi: 10.1371/journal.pone.0195043 29601594 PMC5877860

[8] Goble DJ, Coxon JP, Van Impe A, Geurts M, Van Hecke W, Sunaert S, et al. The neural basis of central proprioceptive processing in older versus younger adults: An important sensory role for right putamen. Hum Brain Mapp. 2012;33(4):895–908. doi: 10.1002/hbm.21257 21432946 PMC6870471

[9] Ingemanson ML, Rowe JB, Chan V, Wolbrecht ET, Cramer SC, Reinkensmeyer DJ. Use of a robotic device to measure age-related decline in finger proprioception. Exp Brain Res. 2016;234(1):83–93. doi: 10.1007/s00221-015-4440-4 26378004 PMC9153390

[10] Lee VMS, Wong TW, Lau CC. Home accidents in elderly patients presenting to an emergency department. Accid Emerg Nurs. 1999 Apr;7(2):96–102. doi: 10.1016/s0965-2302(99)80029-0 10578721

[11] Konczak J, Corcos DM, Horak F, Poizner H, Shapiro M, Tuite P, et al. Proprioception and Motor Control in Parkinson’s Disease. J Mot Behav. 2009 Nov 6;41(6):543–52. doi: 10.3200/35-09-002 19592360

[12] Ingemanson ML, Rowe JR, Chan V, Wolbrecht ET, Reinkensmeyer DJ, Cramer SC. Somatosensory system integrity explains differences in treatment response after stroke. Neurology. 2019;92(10):E1098–108. doi: 10.1212/WNL.0000000000007041 30728310 PMC6442007

[13] Naito E. Sensing Limb Movements in the Motor Cortex: How Humans Sense Limb Movement. Neuroscientist. 2004;10(1):73–82. doi: 10.1177/1073858403259628 14987450

[14] Naito E, Roland PE, Grefkes C, Choi HJ, Eickhoff S, Geyer S, et al. Dominance of the right hemisphere and role of area 2 in human kinesthesia. J Neurophysiol. 2005;93(2):1020–34. doi: 10.1152/jn.00637.2004 15385595

[15] Hagura N, Takei T, Hirose S, Aramaki Y, Matsumura M, Sadato N, et al. Activity in the posterior parietal cortex mediates visual dominance over kinesthesia. J Neurosci. 2007;27(26):7047–53. doi: 10.1523/JNEUROSCI.0970-07.2007 17596454 PMC6672236

[16] Cignetti F, Vaugoyeau M, Nazarian B, Roth M, Anton JL, Assaiante C. Boosted activation of right inferior frontoparietal network: A basis for illusory movement awareness. Hum Brain Mapp. 2014;35(10):5166–78. doi: 10.1002/hbm.22541 24798824 PMC6869717

[17] Meyer S, Kessner SS, Cheng B, Bönstrup M, Schulz R, Hummel FC, et al. Voxel-based lesion-symptom mapping of stroke lesions underlying somatosensory deficits. NeuroImage Clin [Internet]. 2016;10:257–66. Available from: doi: 10.1016/j.nicl.2015.12.005 26900565 PMC4724038

[18] Iandolo R, Bellini A, Saiote C, Marre I, Bommarito G, Oesingmann N, et al. Neural correlates of lower limbs proprioception: An fMRI study of foot position matching. Hum Brain Mapp. 2018;39(5):1929–44. doi: 10.1002/hbm.23972 29359521 PMC6866268

[19] Kenzie JM, Ben-Shabat E, Lamp G, Dukelow SP, Carey LM. Illusory limb movements activate different brain networks than imposed limb movements: an ALE meta-analysis. Brain Imaging Behav. 2018;12(4):919–30. doi: 10.1007/s11682-017-9756-1 28801769

[20] Kenzie JM, Findlater SE, Pittman DJ, Goodyear BG, Dukelow SP. Errors in proprioceptive matching post-stroke are associated with impaired recruitment of parietal, supplementary motor, and temporal cortices. Brain Imaging Behav. 2019;13(6):1635–49. doi: 10.1007/s11682-019-00149-w 31218533

[21] Chilvers MJ, Hawe RL, Scott SH, Dukelow SP. Investigating the neuroanatomy underlying proprioception using a stroke model. J Neurol Sci [Internet]. 2021;430:120029. Available from: doi: 10.1016/j.jns.2021.120029 34695704

[22] Mima T, Sadato N, Yazawa S, Hanakawa T, Fukuyama H, Yonekura Y, et al. Brain structures related to active and passive finger movements in man. Brain. 1999;122(10):1989–97. doi: 10.1093/brain/122.10.1989 10506099

[23] Seiss E, Hesse CW, Drane S, Oostenveld R, Wing AM, Praamstra P. Proprioception-related evoked potentials: Origin and sensitivity to movement parameters. Neuroimage. 2002;17(1):461–8. doi: 10.1006/nimg.2002.1211 12482098

[24] Seiss E, Praamstra P, Hesse CW, Rickards H. Proprioceptive sensory function in Parkinson’s disease and Huntington’s disease: Evidence from proprioception-related EEG potentials. Exp Brain Res. 2003;148(3):308–19. doi: 10.1007/s00221-002-1291-6 12541142

[25] Marini F, Zenzeri J, Pippo V, Morasso P, Campus C. Neural correlates of proprioceptive upper limb position matching. Hum Brain Mapp. 2019;40(16):4813–26. doi: 10.1002/hbm.24739 31348604 PMC6865654

[26] Goble DJ, Lewis CA, Brown SH. Upper limb asymmetries in the utilization of proprioceptive feedback. Exp Brain Res. 2006 Jan 26;168(1–2):307–11. doi: 10.1007/s00221-005-0280-y 16311728

[27] Strong A, Grip H, Arumugam A, Boraxbekk C-J, Selling J, Häger CK. Right hemisphere brain lateralization for knee proprioception among right-limb dominant individuals. Front Hum Neurosci. 2023 Jan 19;17. doi: 10.3389/fnhum.2023.969101 36742357 PMC9892188

[28] Fuentes CT, Bastian AJ. Where Is Your Arm? Variations in Proprioception Across Space and Tasks. J Neurophysiol. 2010 Jan;103(1):164–71. doi: 10.1152/jn.00494.2009 19864441 PMC4116392

[29] van der Helm NA, Gurari N, Drogos JM, Dewald JPA. Task directionality impacts the ability of individuals with chronic hemiparetic stroke to match torques between arms: Preliminary findings. In: 2017 International Conference on Rehabilitation Robotics (ICORR). IEEE; 2017. p. 714–9. 10.1109/ICORR.2017.8009332 28813904

[30] Findlater SE, Desai JA, Semrau JA, Kenzie JM, Rorden C, Herter TM, et al. Central perception of position sense involves a distributed neural network—Evidence from lesion-behavior analyses. Cortex [Internet]. 2016;79:42–56. Available from: doi: 10.1016/j.cortex.2016.03.008 27085894

[31] Ben-Shabat E, Matyas TA, Pell GS, Brodtmann A, Carey LM. The right supramarginal gyrus is important for proprioception in healthy and stroke-affected participants: A functional MRI study. Front Neurol. 2015;6(DEC):1–14. doi: 10.3389/fneur.2015.00248 26696951 PMC4668288

[32] Freyer F, Reinacher M, Nolte G, Dinse HR, Ritter P. Repetitive tactile stimulation changes resting-state functional connectivity-implications for treatment of sensorimotor decline. Front Hum Neurosci. 2012;6(MAY 2012):1–11. doi: 10.3389/fnhum.2012.00144 22654748 PMC3358755

[33] Marini F, Ferrantino M, Zenzeri J. Proprioceptive identification of joint position versus kinaesthetic movement reproduction. Hum Mov Sci [Internet]. 2018;62(March):1–13. Available from: doi: 10.1016/j.humov.2018.08.006 30172030

[34] Hillier S, Immink M, Thewlis D. Assessing Proprioception: A Systematic Review of Possibilities. Neurorehabil Neural Repair. 2015;29(10):933–49. doi: 10.1177/1545968315573055 25712470

[35] Han J, Waddington G, Adams R, Anson J, Liu Y. Assessing proprioception: A critical review of methods. J Sport Heal Sci [Internet]. 2016;5(1):80–90. Available from: doi: 10.1016/j.jshs.2014.10.004 30356896 PMC6191985

[36] Goble DJ, Noble BC, Brown SH. Proprioceptive target matching asymmetries in left-handed individuals. Exp Brain Res. 2009 Aug 2;197(4):403–8. doi: 10.1007/s00221-009-1922-2 19572124

[37] Goble DJ. Proprioceptive Acuity Assessment Via Joint Position Matching: From Basic Science to General Practice. Phys Ther. 2010 Aug 1;90(8):1176–84. doi: 10.2522/ptj.20090399 20522675

[38] Przybyla A, Coelho CJ, Akpinar S, Kirazci S, Sainburg RL. Sensorimotor performance asymmetries predict hand selection. Neuroscience. 2013 Jan;228:349–60. doi: 10.1016/j.neuroscience.2012.10.046 23111126 PMC3714798

[39] Schaefer SY, Haaland KY, Sainburg RL. Hemispheric specialization and functional impact of ipsilesional deficits in movement coordination and accuracy. Neuropsychologia. 2009;47(13):2953–66. doi: 10.1016/j.neuropsychologia.2009.06.025 19573544 PMC2752301

[40] Dempsey-Jones H, Kritikos A. Handedness modulates proprioceptive drift in the rubber hand illusion. Exp Brain Res. 2019 Feb 8;237(2):351–61. doi: 10.1007/s00221-018-5391-3 30411222 PMC6373180

[41] Cohen MX. Analyzing neural time series data: theory and practice. MIT press, editor. 2014.

[42] Burle B, Spieser L, Roger C, Casini L, Hasbroucq T, Vidal F. Spatial and temporal resolutions of EEG: Is it really black and white? A scalp current density view. Int J Psychophysiol. 2015 Sep;97(3):210–20. doi: 10.1016/j.ijpsycho.2015.05.004 25979156 PMC4548479

[43] Kilavik BE, Zaepffel M, Brovelli A, MacKay WA, Riehle A. The ups and downs of beta oscillations in sensorimotor cortex. Exp Neurol. 2013 Jul;245:15–26. 23022918 10.1016/j.expneurol.2012.09.014

[44] Barone J, Rossiter HE. Understanding the Role of Sensorimotor Beta Oscillations. Front Syst Neurosci. 2021 May 31;15. doi: 10.3389/fnsys.2021.655886 34135739 PMC8200463

[45] Tseng Y-T, Chen F-C, Tsai C-L, Konczak J. Upper limb proprioception and fine motor function in young pianists. Hum Mov Sci. 2021 Feb;75:102748. doi: 10.1016/j.humov.2020.102748 33360200

[46] Tseng Y-T, Tsai C-L, Chen F-C. Wrist proprioceptive acuity is linked to fine motor function in children undergoing piano training. J Neurophysiol. 2020 Dec 1;124(6):2052–9. doi: 10.1152/jn.00282.2020 33112691

[47] Landry SP, Champoux F. Musicians react faster and are better multisensory integrators. Brain Cogn. 2017 Feb;111:156–62. doi: 10.1016/j.bandc.2016.12.001 27978450

[48] Lee DN. The functions of vision. Modes of perceiving and processing information. In: Modes of perceiving and processing information. 1st Edition. 1978. p. 159–69.

[49] Elbert T, Pantev C, Wienbruch C, Rockstroh B, Taub E. Increased Cortical Representation of the Fingers of the Left Hand in String Players. Science (80-). 1995 Oct 13;270(5234):305–7. doi: 10.1126/science.270.5234.305 7569982

[50] Scholz J, Klein MC, Behrens TEJ, Johansen-Berg H. Training induces changes in white-matter architecture. Nat Neurosci. 2009 Nov 11;12(11):1370–1. doi: 10.1038/nn.2412 19820707 PMC2770457

[51] Pfurtscheller G, Lopes da Silva FH. Event-related EEG/MEG synchronization and desynchronization: basic principles. Clin Neurophysiol. 1999 Nov;110(11):1842–57. doi: 10.1016/s1388-2457(99)00141-8 10576479

[52] Pfurtscheller G, Neuper C, Pichler-Zalaudek K, Edlinger G, Lopes da Silva FH. Do brain oscillations of different frequencies indicate interaction between cortical areas in humans? Neurosci Lett. 2000 May;286(1):66–8. doi: 10.1016/s0304-3940(00)01055-7 10822154

[53] Goble DJ, Brown SH. Upper Limb Asymmetries in the Matching of Proprioceptive Versus Visual Targets. J Neurophysiol. 2008 Jun;99(6):3063–74. doi: 10.1152/jn.90259.2008 18436632

[54] g.tec. g.HIAMP. https://www.gtec.at/product/ghiamp/. 2022.

[55] Optitrack. Motive Optical motion capture software [Internet]. [cited 2022 Jan 25]. Available from: https://optitrack.com/software/motive/.

[56] Schalk G, Mcfarland DJ, Hinterberger T, Birbaumer N, Wolpaw JR, Technology ABIBCI. BCI2000: A General-Purpose Brain-Computer Interface (BCI) System. 2004;51(6):1034–43. 10.1109/TBME.2004.827072 15188875

[57] Hill NJ, Mooney SWJ, Prusky GT. audiomath: A neuroscientist’s sound toolkit. Heliyon [Internet]. 2021;7(2):e06236. Available from: doi: 10.1016/j.heliyon.2021.e06236 33615015 PMC7881231

[58] Marini F, Squeri V, Morasso P, Konczak J, Masia L. Robot-aided mapping of wrist proprioceptive acuity across a 3D workspace. PLoS One. 2016;11(8):1–12. doi: 10.1371/journal.pone.0161155 27536882 PMC4990409

[59] Peviani V, Bottini G. Proprioceptive errors in the localization of hand landmarks: What can be learnt about the hand metric representation? PLoS One [Internet]. 2020;15(7 July):1–24. Available from: doi: 10.1371/journal.pone.0236416 32735572 PMC7394425

[60] Delorme A, Makeig S. EEGLAB: an open source toolbox for analysis of single-trial EEG dynamics. J Neurosci Methods. 2004;134(1):9–21. 15102499 10.1016/j.jneumeth.2003.10.009

[61] Bigdely-Shamlo N, Mullen T, Kothe C, Su KM, Robbins KA. The PREP pipeline: Standardized preprocessing for large-scale EEG analysis. Front Neuroinform. 2015;9(JUNE):1–19. doi: 10.3389/fninf.2015.00016 26150785 PMC4471356

[62] Christian BJT, Ladd CC, Baecher GB. Artifact Subspace Reconstruction (ASR) for cleaning continuous data. 1995;120(12):2180–207.

[63] Palmer J, Kreutz-Delgado K, Makeig S. AMICA: An Adaptive Mixture of Independent Component Analyzers with Shared Components. San Diego, CA Tech report, Swart Cent Comput Neurosci [Internet]. 2011;1–15. Available from: http://sccn.ucsd.edu/~jason/amica_a.pdf%5Cnpapers2://publication/uuid/E6296FC1-7F6B-400C-85D0-3A292A27F710.

[64] Pion-Tonachini L, Kreutz-Delgado K, Makeig S. ICLabel: An automated electroencephalographic independent component classifier, dataset, and website. Neuroimage. 2019 Sep 1;198:181–97. doi: 10.1016/j.neuroimage.2019.05.026 31103785 PMC6592775

[65] Makeig S. Auditory event-related dynamics of the EEG spectrum and effects of exposure to tones. Electroencephalogr Clin Neurophysiol. 1993 Apr;86(4):283–93. doi: 10.1016/0013-4694(93)90110-h 7682932

[66] Grandchamp R, Delorme A. Single-Trial Normalization for Event-Related Spectral Decomposition Reduces Sensitivity to Noisy Trials. Front Psychol. 2011;2. 21994498 10.3389/fpsyg.2011.00236 PMC3183439

[67] Jain A, Bansal R, Kumar A, Singh K. A comparative study of visual and auditory reaction times on the basis of gender and physical activity levels of medical first year students. Int J Appl Basic Med Res. 2015;5(2):124. doi: 10.4103/2229-516X.157168 26097821 PMC4456887

[68] Introduction Diez P. In: Smart Wheelchairs and Brain-Computer Interfaces. Elsevier; 2018. p. 1–21.

[69] Shelton J, Kumar GP. Comparison between Auditory and Visual Simple Reaction Times. Neurosci Med. 2010;01(01):30–2.

[70] Benjamini Y, Hochberg Y. Controlling the False Discovery Rate: A Practical and Powerful Approach to Multiple Testing. J R Stat Soc Ser B. 1995;57(1):289–300.

[71] Sawilowsky SS. New Effect Size Rules of Thumb. J Mod Appl Stat Methods. 2009 Nov 1;8(2):597–9.

[72] Kimoto Y, Oku T, Furuya S. Neuromuscular and biomechanical functions subserving finger dexterity in musicians. Sci Rep. 2019 Dec 21;9(1):12224. doi: 10.1038/s41598-019-48718-9 31434947 PMC6704118

[73] Graziano MSA. Where is my arm? The relative role of vision and proprioception in the neuronal representation of limb position. Proc Natl Acad Sci. 1999 Aug 31;96(18):10418–21. doi: 10.1073/pnas.96.18.10418 10468623 PMC17903

[74] Touzalin-Chretien P, Ehrler S, Dufour A. Dominance of Vision over Proprioception on Motor Programming: Evidence from ERP. Cereb Cortex. 2010 Aug 1;20(8):2007–16. doi: 10.1093/cercor/bhp271 20026485

[75] Baker SN. Oscillatory interactions between sensorimotor cortex and the periphery. Curr Opin Neurobiol. 2007 Dec;17(6):649–55. doi: 10.1016/j.conb.2008.01.007 18339546 PMC2428102

[76] Crone N. Functional mapping of human sensorimotor cortex with electrocorticographic spectral analysis. I. Alpha and beta event- related desynchronization. Brain. 1998 Dec 1;121(12):2271–99. doi: 10.1093/brain/121.12.2271 9874480

[77] Salmelin R, Forss N, Knuutila J, Hari R. Bilateral activation of the human somatomotor cortex by distal hand movements. Electroencephalogr Clin Neurophysiol. 1995 Dec;95(6):444–52. doi: 10.1016/0013-4694(95)00193-x 8536573

[78] Pfurtscheller G, Stancák A, Neuper C. Event-related synchronization (ERS) in the alpha band—an electrophysiological correlate of cortical idling: A review. Int J Psychophysiol. 1996 Nov;24(1–2):39–46. doi: 10.1016/s0167-8760(96)00066-9 8978434

[79] Rau C, Plewnia C, Hummel F, Gerloff C. Event-related desynchronization and excitability of the ipsilateral motor cortex during simple self-paced finger movements. Clin Neurophysiol. 2003 Oct;114(10):1819–26. doi: 10.1016/s1388-2457(03)00174-3 14499743

[80] Salmelin R, Hari R. Spatiotemporal characteristics of sensorimotor neuromagnetic rhythms related to thumb movement. Neuroscience. 1994 May;60(2):537–50. doi: 10.1016/0306-4522(94)90263-1 8072694

[81] Stancák A, Pfurtscheller G. Event-related desynchronisation of central beta-rhythms during brisk and slow self-paced finger movements of dominant and nondominant hand. Cogn Brain Res. 1996 Oct;4(3):171–83. doi: 10.1016/s0926-6410(96)00031-6 8924046

[82] Engel AK, Fries P. Beta-band oscillations—signalling the status quo? Curr Opin Neurobiol. 2010 Apr;20(2):156–65. doi: 10.1016/j.conb.2010.02.015 20359884

[83] Jasper H, Penfield W. Electrocorticograms in man: Effect of voluntary movement upon the electrical activity of the precentral gyrus. Arch Psychiatr Nervenkr. 1949;163–74.

[84] Schmidt R, Herrojo Ruiz M, Kilavik BE, Lundqvist M, Starr PA, Aron AR. Beta Oscillations in Working Memory, Executive Control of Movement and Thought, and Sensorimotor Function. J Neurosci. 2019 Oct 16;39(42):8231–8. doi: 10.1523/JNEUROSCI.1163-19.2019 31619492 PMC6794925

[85] Zhang Y, Chen Y, Bressler SL, Ding M. Response preparation and inhibition: The role of the cortical sensorimotor beta rhythm. Neuroscience. 2008 Sep;156(1):238–46. doi: 10.1016/j.neuroscience.2008.06.061 18674598 PMC2684699

[86] Tan H, Jenkinson N, Brown P. Dynamic Neural Correlates of Motor Error Monitoring and Adaptation during Trial-to-Trial Learning. J Neurosci. 2014 Apr 16;34(16):5678–88. doi: 10.1523/JNEUROSCI.4739-13.2014 24741058 PMC3988417

[87] Khanna P, Carmena JM. Beta band oscillations in motor cortex reflect neural population signals that delay movement onset. Elife. 2017 May 3;6. doi: 10.7554/eLife.24573 28467303 PMC5468088

[88] van Helvert MJL, Oostwoud Wijdenes L, Geerligs L, Medendorp WP. Cortical beta-band power modulates with uncertainty in effector selection during motor planning. J Neurophysiol. 2021 Dec 1;126(6):1891–902. doi: 10.1152/jn.00198.2021 34731060

[89] Tzagarakis C, Ince NF, Leuthold AC, Pellizzer G. Beta-Band Activity during Motor Planning Reflects Response Uncertainty. J Neurosci. 2010 Aug 25;30(34):11270–7. doi: 10.1523/JNEUROSCI.6026-09.2010 20739547 PMC6633326

[90] Heinrichs‐Graham E, Wilson TW. Coding complexity in the human motor circuit. Hum Brain Mapp. 2015 Dec 25;36(12):5155–67. doi: 10.1002/hbm.23000 26406479 PMC4715608

[91] Heinrichs-Graham E, Wilson TW. Is an absolute level of cortical beta suppression required for proper movement? Magnetoencephalographic evidence from healthy aging. Neuroimage. 2016 Jul;134:514–21. doi: 10.1016/j.neuroimage.2016.04.032 27090351 PMC4912897

[92] Piitulainen H, Seipäjärvi S, Avela J, Parviainen T, Walker S. Cortical Proprioceptive Processing Is Altered by Aging. Front Aging Neurosci. 2018 Jun 14;10. doi: 10.3389/fnagi.2018.00147 29962945 PMC6010536

[93] Walker S, Monto S, Piirainen JM, Avela J, Tarkka IM, Parviainen TM, et al. Older Age Increases the Amplitude of Muscle Stretch-Induced Cortical Beta-Band Suppression But Does not Affect Rebound Strength. Front Aging Neurosci. 2020 May 19;12. 10.3389/fnagi.2020.00117 PMC7248310 32508626

[94] Hammond C, Bergman H, Brown P. Pathological synchronization in Parkinson’s disease: networks, models and treatments. Trends Neurosci. 2007 Jul;30(7):357–64. doi: 10.1016/j.tins.2007.05.004 17532060

[95] Gehringer JE, Arpin DJ, VerMaas JR, Trevarrow MP, Wilson TW, Kurz MJ. The Strength of the Movement-related Somatosensory Cortical Oscillations Differ between Adolescents and Adults. Sci Rep. 2019 Dec 6;9(1):18520. doi: 10.1038/s41598-019-55004-1 31811232 PMC6898653

[96] Haar S, Faisal AA. Brain Activity Reveals Multiple Motor-Learning Mechanisms in a Real-World Task. Front Hum Neurosci. 2020 Sep 2;14. 10.3389/fnhum.2020.00354 PMC7492608 32982707

[97] Pantev C, Engelien A, Candia V, Elbert T. Representational cortex in musicians. Plastic alterations in response to musical practice. Ann N Y Acad Sci. 2001 Jun;930:300–14. 11458837

[98] Wan CY, Schlaug G. Music Making as a Tool for Promoting Brain Plasticity across the Life Span. Neurosci. 2010 Oct 1;16(5):566–77. doi: 10.1177/1073858410377805 20889966 PMC2996135

