# Supplementary material for: Neural correlates of bilateral proprioception and adaptation with training
Source: PLoS One. 2024 Mar 15;19(3):e0299873. doi: 10.1371/journal.pone.0299873 (PMC10942095; doi:10.1371/journal.pone.0299873)
Supplement: S1 Table — (DOCX) [file pone.0299873.s001.docx]

| **ID** | **Age** | **Sex** | **Handedness** | **Instrument** | **experience(yrs)** | **weekly practice(hrs)** |
| --- | --- | --- | --- | --- | --- | --- |
| S04 | 49 | M | R | PIANO | 40 | 12 |
| S06 | 18 | M | R | PIANO | 11 | 12 |
| S10 | 23 | F | R | PIANO | 18 | 8 |
| S11 | 70 | F | R | PIANO | 65 | 3 |
| S12 | 22 | F | R | PIANO | 15 | 20 |
| S17 | 59 | M | R | PIANO | 50 | 15 |
| S18 | 29 | M | R | PIANO | 25 | 7 |
| S23 | 34 | M | R | GUITAR | 21 | 20 |
| S24 | 44 | M | R | PIANO | 33 | 16 |
| **Mean** | 38.7 |  |  |  | 30.9 | 12.6 |
| **STD** | 18.0 |  |  |  | 17.9 | 5.8 |
